# A Low Protein Diet Alters Bone Material Level Properties and the Response to *In Vitro* Repeated Mechanical Loading

**DOI:** 10.1155/2014/185075

**Published:** 2014-08-14

**Authors:** Victor Dubois-Ferrière, René Rizzoli, Patrick Ammann

**Affiliations:** Division of Bone Diseases, Department of Internal Medicine Specialties, University of Geneva Hospitals and Faculty of Medicine, 4 Rue Gabrielle Perret-Gentil, 1211 Geneva 14, Switzerland

## Abstract

Low protein intake is associated with an alteration of bone microstructure and material level properties. However, it remains unknown whether these alterations of bone tissue could influence the response to repeated mechanical loading. The authors investigated the *in vitro* effect of repeated loading on bone strength in humeri collected from 20 6-month-old female rats pair-fed with a control (15% casein) or an isocaloric low protein (2.5% casein) diet for 10 weeks. Bone specimens were cyclically loaded in three-point bending under load control for 2000 cycles. Humeri were then monotonically loaded to failure. The load-displacement curve of the *in vitro* cyclically loaded humerus was compared to the contralateral noncyclically loaded humerus and the influence of both protein diets. Material level properties were also evaluated through a nanoindentation test. Cyclic loading decreased postyield load and plastic deflection in rats fed a low protein diet, but not in those on a regular diet. Bone material level properties were altered in rats fed a low protein diet. This suggests that bone biomechanical alterations consequent to cyclic loading are more likely to occur in rats fed a low protein diet than in control animals subjected to the same *in vitro* cyclic loading regimen.

## 1. Introduction

Malnutrition is frequently observed among older patients [1]. Among nutrients, protein deficiency has been shown to play an important role in the pathogenesis of osteoporosis and fragility fracture Animal studies have shown that an isocaloric low protein intake decreases bone formation [2, 3], leading to a negative bone balance and deterioration of bone mass, bone microstructure, and bone strength in both female and male adult rats [2, 4]. In addition to bone loss and microstructure alterations [5], a low protein diet is associated with changes in bone material level properties [6]. The latter can be detected at the level of the cortical and trabecular bone and is reversible upon protein replenishment [6].

Similar to all materials, cyclic loading of bone leads to a gradual fatigue degradation of mechanical properties over time, resulting from the accumulation of failures or microdamage in bone tissue [7]. As loading continues, microdamages grow in number and size and coalesces until fatigue fractures develop [8–10]. The response of bone to cyclic loading has been previously investigated using different methods including* ex vivo* [8, 9, 11, 12] and* in vivo* protocols [13, 14].* In vivo* techniques take into account bone remodelling responses to cyclic loading [14–17]. The effect of fatigue depends on bone remodelling as well as bone material level properties [14–16]. By contrast,* ex vivo* protocols only assess bone as a material [8, 10].

Alterations of bone material level properties caused by a low protein diet could increase propensity to damage accumulation during cyclic mechanical loading. Therefore, the authors hypothesized that an alteration of bone material level properties caused by a low protein intake could negatively influence the mechanical response of bone to cyclic loading. To test this hypothesis, the authors conducted an* in vitro* study of cyclically loaded bone specimens collected from adult female rats fed either an isocaloric low or a normal protein diet and compared bone strength and material level properties between fatigued and control bones.

## 2. Materials and Methods

### 2.1. Animals and Bone Samples

Six-month-old Sprague-Dawley female rats (Charles River Laboratories, L'Arbresle, France) were housed individually at 21°C with a 12 : 12 h light-dark cycle and strictly pair-fed isocaloric synthetic diets (Provimi Kliba AG, Kaiseraugst, Switzerland) containing 15% or 2.5% casein, 0.8% phosphorus, 1% calcium, 70–80% carbohydrates, and 5% fat throughout the experimental period. The low protein diet was made isocaloric by the addition of corn carbohydrates to ensure the same energy intake for all animals, which also received a daily oral dose of 100 IU/kg of vitamin D dissolved in peanut oil. Demineralized water was available* ad libitum*. As the minimal protein intake necessary for adult rats to maintain bone homeostasis is 5% [2, 3, 18], a restriction to 2.5% corresponds to a reduction of the protein intake of 50% of the necessary amount, a reduction that is often observed in patients with hip fracture [19]. After one week of equilibration on a 15% casein-containing diet, 20 rats were divided into two groups of 10 rats each. Each group received either a 2.5% or a 15% casein-containing diet for 10 weeks. At the end of the experiment, rats were sacrified by an overdose of ketamine hydrochloride. Immediately after sacrifice, the left and right humeri were excised. Specimens were then individually sampled in saline-soaked gauze, sealed in plastic bags, and stored at –20°C until preparation for biomechanical testing. The Animal Ethics Committee of the University of Geneva Faculty of Medicine approved the experimental protocols and all animal procedures.

### 2.2. Bone Geometry: Microcomputed Tomography and Calliper Measurement

For microcomputer tomography (micro-CT) and mechanical tests, humeri pairs were slowly thawed at 7°C overnight, maintained at room temperature, and kept immersed in physiological solution. Each humerus was placed in a plastic tube filled with physiological solution. Bone mass and architecture of the midshaft humerus were analysed in a high-resolution micro-CT system (micro-Ct 40; Scanco Medical, Bassersdorf, Switzerland). A scout-scan and cross-sectional scan were performed on the humerus midshaft, where the load was applied. The mid-diaphysis cortical bone, a 0.168 mm long segment centred over the humerus midline, was scanned at 55 kVp and 145 mA, with resolution of 8-micrometer isotropic voxel size. The resulting grey-scale images were segmented using a low-pass filter to remove noise, and a fixed threshold was employed to extract the mineralized bone phase. The segmentation parameters were set to sigma: 0.8 voxels, support: 1, and threshold: 3.7 cm^−1^. From the binarized images, total volume (TV), bone volume (BV), and cortical bone thickness (C.Th) were calculated by measuring 3D distances, and the structural properties of cortical area (Ct.Ar; mm^2^), moment of inertia (I; mm^4^) were determined. The midshaft humerus diameter (mm) was measured using a calliper with electronic digital display.

### 2.3. Mechanical Testing: Three-Point Bending Test

All mechanical tests were carried out using a servo-controlled electromechanical system (Instron 5566; Instron Corp., High Wycombe, UK) with the actuator displaced at 2 mm/min. Bones were placed in the machine on two supports spaced 16 mm apart with the central loading point situated at the midshaft, thus constituting a three-point bending test [7]. Site of bending was anteroposterior and site of tension was on the posterior surface of the humerus. The tested humerus was fixed in the loading system and enclosed in a small plastic box filled with saline solution. Loading was performed in a saline bath at room temperature (21°C).

**Figure 1:**
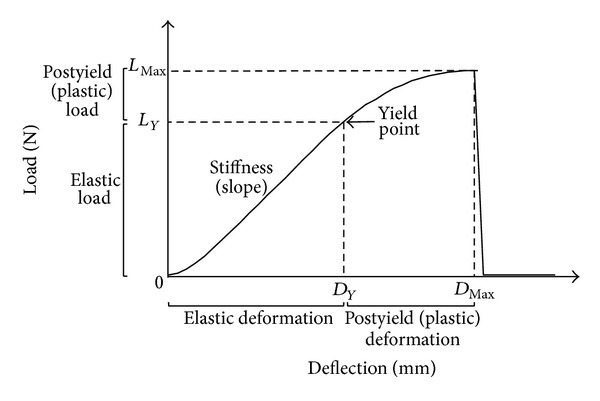
Schematic representation of a load-deflection curve corresponding to a bending test of a bone diaphysis.

Deflection (mm) and load (N) were simultaneously recorded every 0.01 s and the load-deflection curve was recorded (Figure 1). Maximal load (*L*_MAX_ (N)), stiffness (slope of the elastic part of the curve (N/mm)), yield point (*L*_*Y*_, point separating the elastic part and the plastic part of the curve (N)), postyield load (maximal load (*L*_MAX_) minus yield point load (*L*_*Y*_) (N)), and postyield displacement (mm) were directly obtained from the load-deflection curve and automatically calculated. The energy absorbed by the bone tissue (area under the load-deflection curve before the bone breaks (N × mm)), plastic energy (mJ), and elastic energy (mJ) were also calculated.

### 2.4. Fatigue (Cyclic Loading)

#### 2.4.1. Preliminary Determination of Cyclic Loading Parameters

To determine parameters selected for the cyclic loading procedure (cyclic loading at 0.5 Hz under load control for 2000 cycles in a triangular waveform), it has been postulated that the load has to be in the range of the elastic deformation and the number of repetitions should not result in fracture of the bone sample during the cyclic loading period. These parameters were obtained in a pilot study where the humeri of animals fed a normal protein diet were cyclically loaded to failure using different loading conditions. Loading parameter to fatigue was determined from the load-deflection curve of monotonically tested contralateral humerus. Load corresponding to 50%, 60%, 65%, and 70% of the maximal load was applied (number of samples tested = 11) and the mean number of cycles necessary to break the bone was recorded.

#### 2.4.2. Experimental Protocol

Left and right humeri were tested in pairs and randomly allocated to one of two test protocols: monotonic loading or fatigue followed by monotonic loading. The same procedure was applied on humeri of rats fed a normal or a low protein diet. First, one of the two bones was monotonically loaded to failure in a three-point bending test with the actuator displaced at 2 mm/min. Its loading parameters were recorded and the value corresponding to 60% of the maximal load was determined from the load-deflection curve. Secondly, the second humerus was loaded cyclically at 0.5 Hz under load control for 2000 cycles in a triangular waveform. Peak load corresponded to 60% of the maximal load measured from the first bone (contralateral humerus). Finally, the resistance to fracture of the fatigued humerus was determined in a monotonic three-point bending test under the same conditions as the contralateral humerus. Both displacement and load were recorded and the load-displacement curve was determined.

#### 2.4.3. Evaluation of Bending Stress during Cyclic Loading

At the region where the load was applied, the humerus cortical bone is distributed as a cylinder in a quasisymmetrical fashion. Therefore, applied bending stress at the humerus midshaft during fatigue testing was assessed using standard beam theory [7]. Bending stress was determined from the formula *F*∗*L*∗*c*/4*I* where *F* is the applied load, as described above, and corresponding to a load in the linear elastic range; *L* is the span of the loaded specimen, *c* is the cortical radius, and *I* is the moment of inertia, as determined with micro-CT [7].

### 2.5. Bone Material Level Properties: Nanoindentation

Bone material level properties were assessed by nanoindentation. Force-displacements of a pyramidal diamond indenter pressed onto the bone section surface were recorded, using a nanohardness tester (NHT; CSM Instruments, Peseux, Switzerland). This technique has been described in detail elsewhere [6, 20]. In brief, the indenter tip is loaded up to a given depth onto the sample and the load is held constant, leading to a creeping of the material below the tip. Tissue hardness represents the mean pressure the material can resist and is calculated as the ratio of maximum force to contact area. Elastic modulus is defined as the initial slope of the unloading section of the curve. Modulus quantifies the elastic properties of the material and hardness estimates the plastic properties. Both processes could be differently influenced. Dissipated energy corresponds to the area between the loading and unloading curves. The nanoindentation testing was performed on each humerus. The proximal part of transversally cut humeri of each rat was used for nanoindentation. Thus, the tested area was outside the region that was biomechanically either monotonically or cyclically tested. The indentations were centered in the middle of the cortical bone. The samples were embedded in polymethyl methacrylate and the surface of the transverse cut was polished, finishing with a 0.25 *μ*m diamond. Specimens were embedded and cut specimens were then stored at −4°C. Bone samples were rehydrated for 12 h before testing. This procedure decreases the recorded values, suggesting that an integration of water in bone tissue could influence bone material level properties. Therefore, specimens were kept immersed in saline solution throughout the whole analysis. The mechanical tests included five indents on the center of the cortical section of each humerus. A technician blinded to the treatment group performed testing. Indents were done up to 900 nm maximum depth, applying an approximate strain rate of *ε* = 0.066 1/s for both loading and unloading. At maximal load, a 10-second holding period was applied before the hardness parameters were read. The limit of the maximum allowable thermal drift was set to 0.1 nm/s.

### 2.6. Determination of Sample Size

The number of rats was calculated for the primary endpoints: postyield load (N) and postyield deflection (mm). The mean values ± SD typically obtained in rats of this strain and at this age are 20 ± 6.3 (N) and 0.140 ± 0.047 (mm), respectively. From a pilot study, a decrease of 45–60% was expected for each parameter after fatigue (cyclic loading) in rats fed a low protein diet. Therefore, to determine the sample size, a decrease of 45% has been estimated to ensure interpretable results. For a power (90%) and alfa (5%), a sample size of 10 is necessary for the postyield load and 9 for postyield deflection. Thus, the size of the groups was set to 10 rats.

### 2.7. Statistical Analysis

Unpaired Student's *t*-test was performed to compare the effect of cyclic loading on bone strength of humeri between treatment groups. A post hoc power analysis for the measured parameters was performed.

Statistical analysis was performed using Statview software (Statview SE Graphics 1; Abacus Concept, Berkeley, CA). Differences were considered significant at *P* < 0.05. All results are expressed as means ± SEM.

## 3. Results

### 3.1. Preliminary Tests to Determine Experimental Conditions

**Figure 2:**
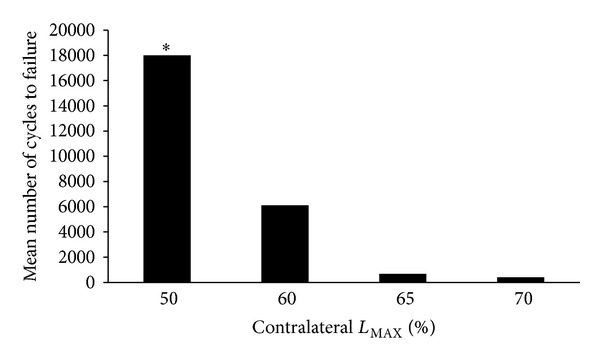
Preliminary determination of cyclic loading. Number of cycles to failure according to different loading parameters based on the maximal load (*L*_MAX_) to failure of the contralateral bone. *Test stopped at 18000 cycles.

Depending on loading forces, bone samples tolerate a different number of cycles before fracture (Figure 2). A loading force corresponding to 60% of maximal force of the contralateral noncyclically loaded humerus allowed a fatigue loading without failure after at least 2000 cycles. However, when increasing the force to higher values, bone rapidly failed. By contrast, a decrease in loading force did not result in failure after more than 18000 cycles. Under the latter condition, only minimal changes would be observed between loaded and nonloaded humeri. Therefore, a loading force of 60% of the maximal load was applied.

### 3.2. Effect of Low Protein Diet on Bone Size, Material Level Properties, and Bone Strength

Midshaft humerus geometry was evaluated by micro-CT in both right and left humeri for each diet group. A statistically significant difference in bone area, total area, and maximum second moment of inertia was observed between the humeri of rats fed a low or a normal protein diet (Table 1). Whole bone resistance to fracture was evaluated on rat humeri monotonically loaded. Maximal load to failure showed a trend to be lower in humeri of rats fed a low protein diet (*P* = 0.91). Stiffness and yield point were unaffected by reducing dietary protein (*P* = 0.32 and *P* = 0.94) (Table 2). Material level properties were tested by nanoindentation. Modulus (−10%; *P* < 0.0001), hardness (−12%; *P* = 0.0003), and dissipated energy (−8.5%; *P* = 0.0001) were significantly lower in rats fed a low protein compared to the control diet (Table 3).

### 3.3. Effect of Cyclic Loading on Bone Mechanical Properties of Humeri of Rats Fed a Normal or a Low Protein Diet

In rats fed a normal protein diet, mechanical properties were almost unaffected by cyclic loading (Table 2) and maximal load to failure and stiffness remained unchanged. Similarly, postyield load and plastic deflection were not modified by fatigue loading (Figure 2), including plastic energy (Table 2).

**Figure 3:**
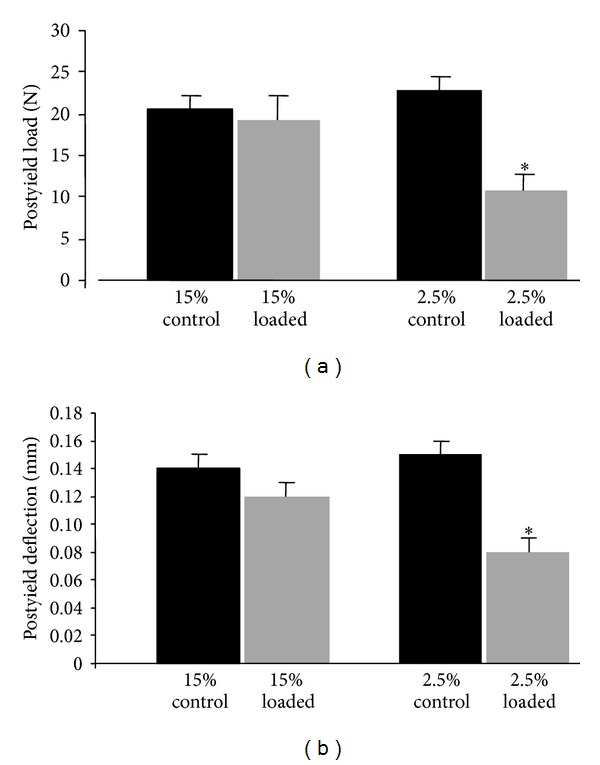
Postyield load and deflection in monotonically loaded humerus of 6-month-old rats fed either normal casein (15%) or low casein diet (2.5%). Humeri were either previously cyclically loaded (open columns) or served as control (dark columns). Values are means ± SEM. *P < 0.05 versus 2.5% casein diet.

In rats fed a low protein diet, maximal load and stiffness showed a trend to a decrease but it was not statistically significant after cyclic loading (Table 2). However, postyield load and plastic deflection were both significantly decreased (−45% and −30%, resp.) after loading, resulting in a significant decrease of plastic energy (−38%) (Figure 3).

### 3.4. Post Hoc Power Analysis

A post hoc power analysis was performed for the measured parameters used for statistical comparisons. The following values were obtained: maximal load, *n* = 15; stiffness, *n* = 254; plastic energy, *n* = 8; postyield load, *n* = 5; postyield deflection, *n* = 7.

## 4. Discussion

In this study, bone strength was not significantly changed by the low protein diet. These results contrast with previous studies, demonstrating a reduction of bone strength in rats fed a low protein diet and a correction when essential amino acids supplements were given [5, 18]. However, the duration of protein restriction was shorter in our study, 10 weeks instead of 18 weeks previously reported [3, 21]. A lack of a significant effect was also observed in another study in which a 4-week protein restriction did not alter bone strength [3]. Nanoindentation tests showed a significant change of bone material level properties in animals fed a low protein diet. After a period of only 10 weeks, an alteration of bone material level properties was already observed in rats fed a low protein diet. This early degradation of bony tissue has been also previously observed in rats [6] and mice [22] and an alteration of bone material level properties could represent the initial bone degradation process in a situation of reduced protein intake.

After cyclic loading, bone biomechanical properties were altered in rats fed an isocaloric low protein diet, but not in control rats. Alterations of bone biomechanical properties were mostly observed in the plastic phase of load-displacement curve as illustrated by a significant reduction of postyield load, postyield deflection, and plastic energy. In the load-deflection curve, the postyield or non-Hookean behavior corresponds to the phase beyond the yield point where the bone deforms nonlinearly with the load [7]. During this phase, deformations are irreversible and result from small microfractures and crack dissemination. The accumulation of microcracks seems also to play a role in alteration of postyield behaviour as observed in older bone [23]. In the present study, the degradation of parameters reflecting plastic behavior in rats fed a low protein diet after cyclic loading could be explained by an alteration of bone material level properties, leading potentially to an increased propensity to damage accumulation.

Results of bone material level properties using nanoindentation highlight the major deleterious effects of a low protein diet on humerus cortical bone quality, which have been already observed in previous studies [6]. Altered bone material level properties certainly play important roles in the modifications of postyield variables after cyclic loading in rats fed a low protein diet. The authors suggest that these alterations could facilitate the appearance of damage and microcracks. Unfortunately, it was not possible to evaluate the number of microcracks in the present study as both humeri were used in three-point bending tests after cyclic loading.

To the best of the authors' knowledge, this is the first study comparing the effects of protein restriction on bone fatigue. A 10-week period of low protein intake was chosen to limit the effects of such a diet on bone geometry and microstructure and to assess mainly the material level properties of bone. In the case of a longer period of low protein intake, alterations of bone architecture have been shown [21] and conditions of cyclic loading may have been too different between both groups. A post hoc power analysis was performed and confirmed that the selected number of rats was adequate to assess the postyield parameters, which are the primary endpoints of the current study. It is not surprising that the number of rats was insufficient to assess other parameters, such as stiffness and maximal load, as it is known that these parameters take longer time to change in response to low protein diet.

Malnutrition or undernutrition, particularly protein malnutrition, is frequently observed among the elderly. Previous studies have shown that isocaloric low protein intake is associated with decreased bone mass and bone strength in adult female and male rat, as well as major alterations of bone microarchitecture and decreased intrinsic bone tissue quality in rats [2, 3, 6, 18, 21]. In this study, low protein intake is associated with an altered response to cyclic loading and this could participate in the higher propensity to fracture observed in case of malnutrition or undernutrition. However, this study assesses an* ex vivo* response and further studies should investigate* in vitro* response to cyclic loading in case of low protein intake.

An* in vitro* cyclic loading allowed the authors to test the effects of fatigue independent of a biologic or muscular response. The current study's protocol included an evaluation of bone strength after cyclic loading during a monotonic loading to failure to compare the different phases of load-displacement curve between control and cyclic loading conditions. This model could be used also for testing the efficacy of pharmacological interventions against osteoporosis and other characteristics of biomechanics. However, the current model does not take into account the remodeling process induced by fatigue loading of bone, which has been shown in* in vivo* studies [24]. This microdamage repair process should also be taken into account when evaluating the efficacy of drugs. Indeed, the effect of drugs on remodeling could alter the response of bone to fatigue. For example, bisphosphonate therapy can inhibit remodeling, through inhibition of osteoclast function. This can impair the repair of microdamage and lead to accumulation of bone microdamage.

## 5. Conclusion

In summary, alterations of bone material level properties in rats fed a low protein diet are associated with alterations of postyield behavior. The bone of a rat fed a low protein diet may be more likely to fatigue under the same loading conditions than the bones of a rat fed a normal protein diet.

## Figures and Tables

**Table 1:** Humerus bone geometry determined by *μ*CT in rats fed a normal or a low protein diet.

	Dietary protein	
	15%	2.5%
Bone area (mm^2^)	3.95 ± 0.03*	3.76 ± 0.04
Total area (mm^2^)	4.86 ± 0.06*	4.65 ± 0.06
*I*_max⁡_ (mm^4^)	2.27 ± 0.06*	2.00 ± 0.05
*I*_min⁡_ (mm^4^)	1.53 ± 0.05	1.42 ± 0.04
Estimated bending stress (MPa)	921 ± 71.3	728.4 ± 61.9

Values are means ± SEM.

*P < 0.05 versus.

2.5% casein diet.

**Table 2:** Effect of cyclic loading on humerus bone strength in rats fed a normal or a low protein diet.

	Dietary protein			
	15% controls	15% contralateral cyclically loaded	2.5% controls	2.5% contralateral cyclically loaded
Maximal load (N)	92.3 ± 2.9	90.8 ± 2.8	87.1 ± 2.5	77.8 ± 4.8
Stiffness (N/mm)	236.6 ± 16.5	272.8 ± 17.9	237.5 ± 12.4	218.6 ± 22.5
Yield point (N)	71.8 ± 3.6	71.4 ± 1.6	64.4 ± 2.7	67.1 ± 3.3
Maximal energy (N∗mm)	24.2 ± 1.4	20.6 ± 1.0*	20.8 ± 0.7	17.9 ± 1.2
Elastic energy (N∗mm)	12.3 ± 1.5	10.4 ± 0.8	9.1 ± 0.8	11.4 ± 0.8
Plastic energy (N∗mm)	11.9 ± 0.9	10.3 ± 1.4	11.7 ± 0.7	6.6 ± 1.2°

Values are means ± SEM.

*P < 0.05 versus 15% controls as evaluated by Student's *t*-test.

°*P* < 0.05 versus 2.5% controls as evaluated by Student's *t*-test.

**Table 3:** Effect of low protein diet on proximal humerus cortical material level properties.

	Dietary protein	
	15%	2.5%
Modulus (gPa)	19.39 ± 0.33	17.44 ± 0.25*
Hardness (mPa)	777.4 ± 16.3	684.9 ± 16.3*
Dissipated energy (mN∗nm)	4461.0 ± 70.4	4082.1 ± 70.7*

Values are means ± SEM.

*P < 0.05 versus 15% as evaluated by Student's *t*-test.

## References

[1] Smith TK (1991). Nutrition: its relationship to orthopedic infections. *Orthopedic Clinics of North America*.

[2] Bourrin S, Ammann P, Bonjour JP, Rizzoli R (2000). Dietary protein restriction lowers plasma insulin-like growth factor I (IGF-I), impairs cortical bone formation, and induces osteoblastic resistance to IGF-I in adult female rats. *Endocrinology*.

[3] Bourrin S, Toromanoff A, Ammann P, Bonjour JP, Rizzoli R (2000). Dietary protein deficiency induces osteoporosis in aged male rats. *Journal of Bone and Mineral Research*.

[4] Ammann P, Rizzoli R (2003). Bone strength and its determinants. *Osteoporosis International*.

[5] Ammann P, Laib A, Bonjour J-, Meyer JM, Rüegsegger P, Rizzoli R (2002). Dietary essential amino acid supplements increase bone strength by influencing bone mass and bone microarchitecture in ovariectomized adult rats fed an isocaloric low-protein diet. *Journal of Bone and Mineral Research*.

[6] Hengsberger S, Ammann P, Legros B, Rizzoli R, Zysset P (2005). Intrinsic bone tissue properties in adult rat vertebrae: modulation by dietary protein. *Bone*.

[7] Turner CH, Burr DB (1993). Basic biomechanical measurements of bone: a tutorial. *Bone*.

[8] Burr DB, Turner CH, Naick P (1998). Does microdamage accumulation affect the mechanical properties of bone?. *Journal of Biomechanics*.

[9] Danova NA, Colopy SA, Radtke CL (2003). Degradation of bone structural properties by accumulation and coalescence of microcracks. *Bone*.

[10] Hoshaw SJ, Cody DD, Saad AM, Fyhrie DP (1997). Decrease in canine proximal femoral ultimate strength and stiffness due to fatigue damage. *Journal of Biomechanics*.

[11] Les CM, Stover SM, Keyak JH, Taylor KT, Kaneps AJ (2002). Stiff and strong compressive properties are associated with brittle post-yield behavior in equine compact bone material. *Journal of Orthopaedic Research*.

[12] Cointry GR, Capozza RF, Negri AL, Ferretti JL (2005). Biomechanical impact of aluminum accumulation on the pre- and post-yield behavior of rat cortical bone. *Journal of Bone and Mineral Metabolism*.

[13] Uthgenannt BA, Silva MJ (2007). Use of the rat forelimb compression model to create discrete levels of bone damage in vivo. *Journal of Biomechanics*.

[14] Bentolila V, Boyce TM, Fyhrie DP, Drumb R, Skerry TM, Schaffler MB (1998). Intracortical remodeling in adult rat long bones after fatigue loading. *Bone*.

[15] Robling AG, Hinant FM, Burr DB, Turner CH (2002). Improved bone structure and strength after long-term mechanical loading is greatest if loading is separated into short bouts. *Journal of Bone and Mineral Research*.

[16] Hsieh Y, Silva MJ (2002). In vivo fatigue loading of the rat ulna induces both bone formation and resorption and leads to time-related changes in bone mechanical properties and density. *Journal of Orthopaedic Research*.

[17] Warden SJ, Hurst JA, Sanders MS, Turner CH, Burr DB, Li J (2005). Bone adaptation to a mechanical loading program significantly increases skeletal fatigue resistance. *Journal of Bone and Mineral Research*.

[18] Ammann P, Bourrin S, Bonjour J, Meyer J, Rizzoli R (2000). Protein undernutrition-induced bone loss is associated with decreased IGF-I levels and estrogen deficiency. *Journal of Bone and Mineral Research*.

[19] Delmi M, Rapin CH, Bengoa JM, Delmas PD, Vasey H, Bonjour JP (1990). Dietary supplementation in elderly patients with fractured neck of the femur. *The Lancet*.

[20] Ammann P, Badoud I, Barraud S, Dayer D, Rizzoli R (2007). Strontium ranelate treatment improves trabecular and cortical intrinsic bone tissue quality, a determinant of bone strength. *Journal of Bone and Mineral Research*.

[21] Mekraldi S, Toromanoff A, Rizzoli R, Ammann P (2005). Pamidronate prevents bone loss and decreased bone strength in adult female and male rats fed an isocaloric low-protein diet. *Journal of Bone and Mineral Research*.

[22] Brennan-Speranza TC, Rizzoli R, Kream BE, Rosen C, Ammann P (2011). Selective osteoblast overexpression of IGF-I in mice prevents low protein-induced deterioration of bone strength and material level properties. *Bone*.

[23] Courtney AC, Hayes WC, Gibson LJ (1996). Age-related differences in post-yield damage in human cortical bone. Experiment and model. *Journal of Biomechanics*.

[24] Wu B, Zhang C, Chen B (2013). Self-repair of rat cortical bone microdamage after fatigue loading in vivo. *International Journal of Endocrinology*.

